# Retraction Note: Long non-coding RNA PTPRG-AS1 promotes cell tumorigenicity in epithelial ovarian cancer by decoying microRNA-545-3p and consequently enhancing HDAC4 expression

**DOI:** 10.1186/s13048-023-01123-3

**Published:** 2023-03-01

**Authors:** Juanjuan Shi, Xijian Xu, Dan Zhang, Jiuyan Zhang, Hui Yang, Chang Li, Rui Li, Xuan Wei, Wenqing Luan, Peishu Liu

**Affiliations:** 1 Department of Gynaecology, Qilu Hospital, Cheeloo College of Medicine, Shandong University, 107 West Wenhua Road, Jinan, 277599 Shandong China; 2 Department of Gynaecology, Tengzhou Center People’s Hospital, Zaozhuang, 277500 Shandong China; 3 Department of Gynaecology, Rizhao Central Hospital, Rizhao, 276800 Shandong China; 4 Department of TCM Pharmacy, Tengzhou Center People’s Hospital, Zaozhuang, 277500 Shandong China; 5 Department of Clinical Pharmacy, Tengzhou Center People’s Hospital, Zaozhuang, 277500 Shandong China; 6 Department of Pathology, Tengzhou Center People’s Hospital, Zaozhuang, 277500 Shandong China

**Retraction Note: J Ovarian Res 13, 127 (2020)**

10.1186/s13048-020-00723-7

The Editors-in-Chief have retracted this article. After publication, concerns were raised regarding the flow cytometry plots presented in the article. Specifically:

In Fig. 3c, the two miR-545-3p mimic panels appear highly similar.

Fig. 5c OVCAR3 middle panel appears highly similar to Fig. 6c MG-63 middle panel in [1].

Fig. 6C OVCAR3 middle panel appears highly similar to Fig. 4b MG-63 right panel in [1].

The two articles also exhibit highly similar study design, data presentation style and western blot appearance despite being written by different author groups.

The authors have stated that they uploaded the wrong images from third party equipment. The Editors-in-Chief therefore no longer have confidence in the presented data.

None of the authors have responded to any correspondence from the editor or publisher about this retraction notice.
